# The measurement of reactive oxygen species in human neat semen and in suspended spermatozoa: a comparison

**DOI:** 10.1186/1477-7827-7-118

**Published:** 2009-10-27

**Authors:** Helena Fingerova, Ivana Oborna, Jiri Novotny, Magda Svobodova, Jana Brezinova, Lenka Radova

**Affiliations:** 1 Department of Obstetrics and Gynecology, University Hospital Olomouc, IP Pavlova 6, 775 20 Olomouc, Czech Republic

## Abstract

**Background:**

It is generally accepted that oxidative stress is an important factor in male infertility because it may impair the physiological function of spermatozoa at the molecular level. Nevertheless, although several approaches have been reported, the imbalance between production of reactive oxygen species (ROS) and activity of the antioxidant defense system in semen is difficult to investigate and remains poorly understood.

**Methods:**

This study compares measurement of ROS production in neat semen and in washed spermatozoa obtained from the same ejaculate, and suspended in phosphate buffered saline using exactly the same luminol-mediated chemiluminescence method. Ninety one samples were obtained from males of infertile couples and 34 from volunteers with proven fertility.

**Results:**

As expected, ROS levels were markedly lower in neat semen than in washed spermatozoa suspensions where seminal plasma with its potent antioxidant capacity was removed. In the cases of both neat semen and washed spermatozoa, ROS production was lowest in samples from normozoospermic males and highest in samples containing more than half million peroxidase-positive leukocytes per milliliter. For all samples, there was a significant positive correlation between ROS production by neat semen and that by washed spermatozoa suspension.

**Conclusion:**

Measurement of ROS production in neat semen better reflects actual oxidative status because it detects only the overproduction of ROS which are not effectively scavenged by antioxidant capacity of seminal fluid. The results of our study show a good commutability of both measurements for identification of semen samples with high ROS production. The measurement in neat semen is even less time consuming and therefore easier to implement into laboratory routine.

## Background

Reactive oxygen and reactive nitrogen species (ROS and RNS), a group of highly reactive oxidants, most of which contain unpaired electrons, are usually known as ROS or free radicals. At physiological levels they have important roles in metabolism in all aerobic organisms. However, excessive ROS production which can not be effectively controlled by antioxidants leads to oxidative stress (OS) which has been linked to many pathological processes, including male infertility [1-3]. In the genital tract, low levels of ROS are necessary for normal function of human spermatozoa [4], including their capacitation, acrosome reaction and sperm-oocyte fusion. On the other hand, excessive OS may cause lipoperoxidation of sperm membranes resulting in DNA damage and sperm apoptosis. Standard semen analysis is often inadequate to explain conception failure as the routine microscopic evaluation can not reveal subtle disorders at the molecular level which may be caused by OS [5]. This situation often leads to diagnosis of idiopathic infertility. Moreover, there is some degree of overlap in sperm parameters between fertile and infertile males [6].

Although the importance of seminal OS assessment has already been advocated for semen analysis by WHO Laboratory Manual 1999 [7], only a few centers worldwide have so far developed methods for indirect measurement of ROS production in semen. ROS production in human spermatozoa was first measured in washed spermatozoa suspended in phosphate buffered saline (PBS) to the concentration of 20 millions per milliliter. The chemiluminescent signal induced by the addition of luminol was measured integrally and expressed in cpm [2,8,9]. We implemented this method in the University Hospital in Olomouc in 2002, to our knowledge for the first time in the Czech Republic. The weakness of this approach is that seminal plasma with its powerful total antioxidant capacity (TAC) is removed prior to measurement. Later, as a better predictor of oxidative sperm damage, the ROS/TAC score was introduced [10]. Unfortunately, both ROS and TAC measurements are subject to various methodological problems. ROS measurement by luminol-induced chemiluminescence in neat semen was first reported by Allamaneni *et al*. [11]. Since the beginning of 2007 we have been measuring ROS simultaneously in neat ejaculate and in spermatozoa suspension in PBS, i.e. washed semen. The aim of our study was to compare their clinical usefulness in the evaluation of the male factor infertility and to evaluate the commutability between the ROS measurement in washed and neat semen.

## Methods

### Study group

Within the period of two years, 91 semen samples were obtained from 85 males from couples asking for enrollment into infertility treatment. The only inclusion criterion was a sperm concentration over 20 × 10^6^/ml to ensure a sufficient precision of ROS measurement. Another 34 semen samples from 22 volunteers with proven fertility served as a control group. The time span between the repeated semen samples in 18 subjects was at least 6 months and in this sense was considered as an independent variable. The average age of all subjects was 33 ± 5 years and there was no significant difference in age between the evaluated groups. The study was approved by the Ethical Board of the Palacky University Medical Faculty in Olomouc and all subjects signed written consent.

### Study design

Ejaculates were obtained by masturbation following three to four days of sexual abstinence. After liquefaction (37°C, 30 min), standard semen analysis was performed according to the WHO Manual [7]. One aliquot of 0.5 ml of liquefied neat semen was used for immediate ROS measurement and another 1 ml aliquot of semen was taken for preparation of spermatozoa suspension in PBS and following ROS measurement by the same luminol-induced chemiluminescence method [12].

### ROS detection by chemiluminescent assay in sperm suspension in PBS

The method was described in detail previously [12]. Briefly, the liquefied semen was centrifuged at 300 g for 7 min, seminal plasma was removed and the pellet of cells was washed in PBS (isotonic solution, pH = 7.4) and spun again and decanted. Washed cells were suspended in PBS to adjust sperm concentration to 1.25 × 10^6^/ml. ROS production was measured after addition of 10 μl of 5 mM freshly prepared solution of luminol (5-amino-2,3-dihydro-1,4-phthalazinedione, Sigma Chemical Co., St. Louis, MO, USA) in dimethylsulphoxide (DMSO, Sigma Chemical Co.) to 400 μl of spermatozoa suspension. A tube containing 400 μl of PBS and 10 μl of luminol solution served as a blank. Chemiluminescence was measured integrally for 15 minutes using the Digene DCR-1 single detector luminometer (Digene Diagnostics, Inc., Gaithersburg, MD, USA). Results were expressed in relative light units (RLU) per minute and per 20 × 10^6 ^spermatozoa.

### ROS detection by chemiluminescent assay in neat semen

The ROS production in 400 μl of liquefied neat semen was measured after addition of 10 μl of 5 mM solution of luminol in DMSO. A tube containing 10 μl of 5 mM luminol solution in DMSO was used as a blank. Chemiluminescence was measured for 15 min as described above. The RLU/min were then recalculated according to the original spermatozoa concentration in semen sample and expressed as RLU/min per 20 × 10^6 ^spermatozoa.

### Statistical analysis

Statistical calculations were performed using Statistica 8 (StatSoft CR) [13]. Mann-Whitney test was used to compare data between groups. Spearman correlation coefficient was used to evaluate interdependence between the ROS production found in neat semen and that found in sperm suspension. Logarithmic transformation was used for this purpose due to a very large range of inter-individual values. Statistical significance was set at p < 0.05 for all tests.

## Results

### Semen analysis

Semen samples were classified according to the WHO Manual [7] as normozoospermic when sperm concentration was ≥ 20 × 10^6^/ml, motility ≥ 50%, morphology ≥ 30% and Endtz test <1.0 × 10^6^/ml [14]. On the basis of microscopic evaluation, 39 semen samples from males of infertile couples were normozoospermic (NS-males) and 52 semen samples (SA-males) revealed various semen abnormalities which included 14 asthenozoo-, 29 astenoteratozoo-, 4 teratozoo- and 5 samples with leukocyte count over 0.5 × 10^6^/ml including three leukocytospermias. Semen samples (n = 34) of fertile volunteers (FV-males) served as a control. With regard to our previous finding [15] that a concentration of peroxidase positive leukocytes in Endtz test higher than 0.5 × 10^6^/ml may significantly contribute to ROS production in semen, we have further subdivided the SA group into Endtz test negative samples (SA EN subgroup, n = 27), samples with leukocyte concentrations below 0.5 × 10^6^/ml (SA EL subgroup, n = 20) and over 0.5 × 10^6^/ml (SA EH subgroup, n = 5). The data on age and semen parameters of all subjects enrolled in the study are shown in Table 1.

**Table 1:** Semen parameters of all subjects

**Number of samples**	**FV** **n = 34**	**NS** **n = 39**	**SA** **n = 52**
Semen volume (ml)	2.8 ± 1.3	3.2 ± 1.4	3.3 ± 1.3
Total sperm count (× 10^6^)	169 ± 104	195 ± 99	155 ± 77
Sperm concentration (× 10^6^/ml)	64.5 ± 29.7	65.9 ± 31.1	50.9 ± 25.0
Motility (%)	44.2 ± 10.2	53.8 ± 7.7	37.3 ± 8.8
Normal morphology (%)	31.5 ± 8.6	38.1 ± 5.8	25.1 ± 8.2

FV - fertile volunteers, NS - normozoospermic males, SA - males with semen abnormalities.

Values are expressed as mean ± SD

### ROS detection in neat semen

As seen in Table 2, the lowest ROS production in neat semen was found in the fertile volunteers (FV), as well as in normozoospermic males from infertile couples (NS) and in leukocyte-free samples from males with semen abnormalities (SA EN). Significantly higher ROS production compared to fertile volunteers was found in SA EL samples (p < 0.005) and the highest ROS production was found in SA EH samples (p < 10^5^).

**Table 2:** Differences of ROS production in neat semen and in spermatozoa suspension between fertile volunteers and subgroups of males from infertile couples

**groups**	**n**	**ROS (RLU × 10^3^) in neat semen**	**^a^p**	**ROS (RLU × 10^4^) in spermatozoa suspension**	**^b^p**
FV	34	0.26 (0.12; 0.55)		3.5 (1.3; 13.0)	
NS	39	0.31 (0.12; 1.12)	<0.52	2.4 (1.0; 20.2)	<0.91
SA	52	1.1 (0.19; 4.1)	<0.005	9.3 (1.4; 118.3)	<0.055
SA subgroups of SA EN	27	0.24 (0.15; 1.30)	<0.27	3.9 (0.9; 28.2)	<0.1
SA EL	20	1.14 (0.36; 4.2)	<0.005	11.0 (2.0; 107.3)	<0.76
SA EH	5	32.3 (7.40; 55.8)	<10^-5^	385.0 (170.0; 625.0)	<10^-5^

Values are presented as median (25^th^; 75^th ^percentile). The ^a^p and ^b^p values designate the differences in ROS production in subgroups of males from infertile couples versus FV in neat semen and in spermatozoa in PBS suspension, respectively. A value of p < 0.05 was considered significant by Mann-Whitney *U *test.

FV - fertile volunteers, NS - normozoospermic males, SA - males with semen abnormalities,

SA EN - subgroup of SA with negative Endtz test, SA EL - subgroup of SA with Endtz test <0.5 × 10^6^/ml, SA EH - subgroup of SA with Endtz test >0.5 × 10^6^/ml

### ROS detection in sperm suspension in PBS

ROS production in sperm suspension was generally higher than that in neat semen (up to 1 × 10^7 ^RLU/min per 20 × 10^6 ^spermatozoa). There was no difference in ROS production between FV, NS, SA EN and SA EL males. A significant difference (p < 10^-5^) was found only in the SA EH group.

**Figure 1:**
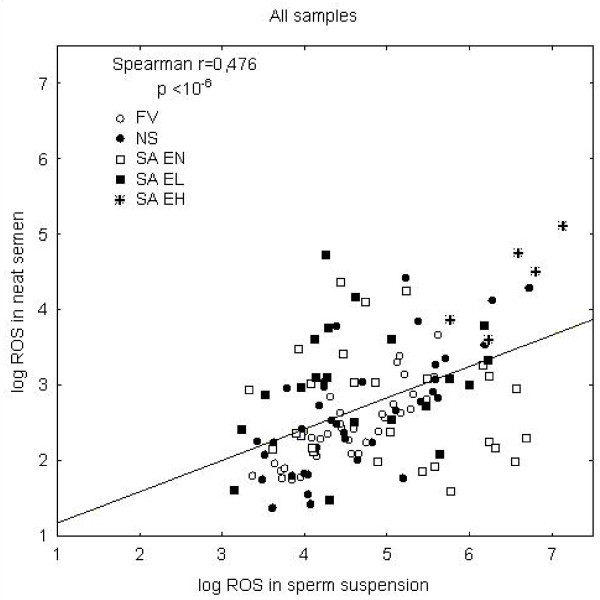
**Correlation between log_10 _ROS in neat semen and log_10 _ROS in sperm suspension in PBS in all samples**. Individual groups are differentiated by symbols.

There was a significant positive correlation (r = 0.476, p < 1 × 10^-6^) between the ROS levels in neat semen and those in sperm suspension (Fig 1). Most of the FV and NS samples were closer to the regression line and prevalently below it, while the SA samples were much more widely scattered along the regression line in both directions. On the contrary, the SA EH samples were all above the regression line.

## Discussion

Since the appearance of the first reports in the early nineteen nineties [1,2,16,17], the role of OS in the pathophysiology of male infertility has been gradually accepted. The importance of seminal ROS production has been already stressed in the WHO Manual (1999) and several methods of ROS detection in semen have been reported [8,9,12,18]. Nevertheless, reliable and reproducible methods of ROS measurement in semen for routine clinical use are still missing. Such method(s) would be a useful tool in the diagnosis of male infertility and in the selection of patients who would benefit from antioxidant treatment [3,18-22].

Measurement of the level of OS in ejaculate arising from imbalance between ROS production and the capacity of the complex antioxidant defense system is extremely difficult. The direct measurement of free radicals is practically impossible. The indirect methods have either used lucigenin or luminol mediated chemiluminescence in spermatozoa suspension [1], or they have to rely on the detection of some stable oxidized end-products, the so called biomarkers of OS, mostly in body fluids [23]. Luminol mediated luminescence is preferred as it can detect the sum of several important intra- and extra-cellular ROS including hydrogen peroxide, superoxide and hydroxyl radicals [2,9,16].

Unfortunately, there are some limitations to ROS measurement in spermatozoa suspension in PBS. The absolute values are expressed either in count per minute (cpm) or RLU per minute, and moreover, may differ with respect to the sensitivity and type of luminometer used. ROS production by spermatozoa suspended in PBS declines with time, therefore the measurement should be performed within one hour of obtaining semen sample [9]. There is also a possibility of an artificial increase in ROS production caused by repeated centrifugation during the preparation of the spermatozoa suspension [14]. All this makes it difficult to compare results reported from different settings. Another, even more important drawback of the measurement of ROS in washed spermatozoa, is the fact that they are deprived of their natural antioxidant environment, seminal plasma. The ROS/TAC score [10], thought to assess only the excess of ROS not scavenged by seminal plasma antioxidants, introduces another variable which may also be prone to analytical error. According to our experience [24], the TAC of seminal plasma measured by the TAS Randox^® ^method varied to a much smaller extent in contrast to ROS production because it measured only non-enzymatic antioxidants.

The determination of ROS production in neat semen is a better solution. It avoids centrifugation and washing procedures and shortens the time lag between semen collection and ROS measurement. Moreover, it can be expected to measure only the excess ROS which are not scavenged by seminal plasma antioxidants and thus directly identify samples with OS. In our setting, the ROS levels in neat semen were lowest in samples from fertile volunteers, normozoospermic men and leukocyte-free samples from men with semen abnormalities. ROS levels were significantly higher in samples with peroxidase-positive leukocyte concentrations <0.5 × 10^6^/ml and the highest in samples with leukocyte concentrations >0.5 × 10^6^/ml. The corresponding results measured in spermatozoa suspension in PBS were markedly higher, in some cases up to two orders of magnitude. A significantly higher ROS production was detected only in samples from SA males with leukocyte concentrations >0.5 × 10^6^/ml.

Our findings of a reasonable commutability of measuring ROS in washed and neat semen well agree with the first reported comparison of luminol-mediated chemiluminescent measurement of ROS production in neat semen versus spermatozoa suspensions in PBS [11]. Allamameni *et al*. evaluated semen from 34 semen donors and 44 patients with abnormal semen parameters. The ROS production in neat semen of donors was about five times lower than the respective ROS levels in spermatozoa suspension. The levels measured in neat semen of patients with abnormal semen were significantly higher than in healthy donors. A later study of Athayde *et al*. included semen samples from 114 fertile Brazilian men seeking voluntary sterilization by vasectomy and 47 subfertile males [25]. In samples without leukocytes, the threshold for normality of ROS in neat semen samples was set at 0.55 × 10^4 ^cpm per 20 × 10^6 ^spermatozoa; almost 20 times lower than that in washed spermatozoa in PBS. A recent study by this group [26] reported the median and interquartile range of seminal ROS levels in neat semen samples of 78 vasectomy candidates younger than 40 years as 0.29 (0.18, 0.58) × 10^4 ^cpm per 20 × 10^6 ^spermatozoa, which agrees well with our findings in Czech fertile volunteers, that is 0.26 (0.12, 0.55) × 10^3 ^RLU/min per 20×10^6 ^spermatozoa, even though they were obtained on a different population and using a different luminometer.

This study, to our knowledge the first one in the Czech Republic, has proved that a significant positive correlation exists between ROS levels in neat semen and that in spermatozoa suspension in PBS, in which the antioxidant capacity of seminal plasma is absent. This suggests that the individual total antioxidant capacity of seminal plasma may vary to a lesser extent than the ROS produced by spermatozoa and/or activated leukocytes.

## Conclusion

We conclude that ROS measurement by luminol-mediated chemiluminescence in neat semen provides a better assessment of the actual level of OS than the measurement in washed spermatozoa. The ROS determination in neat semen is rapid, simple and can be used also for ROS measurement in severe oligozoospermia [27]. Though the role of OS as a cause of male infertility has been generally accepted, ROS measurement has not yet been routinely used in clinical infertility treatment. There is still a lack of a single standardized measure of OS [3]. Laboratories which want to introduce OS evaluation by this chemiluminescence method will have to establish and validate their own reference ranges. Further research is also needed to elucidate if and how ROS production in neat semen correlates with other surrogate OS markers [3,23].

## Competing interests

The authors declare that they have no competing interests.

## Authors' contributions

HF wrote the paper, IO designed the study, recruited study subjects and controls, provided clinical information and gave final approval, JN perfomed chemiluminesce measurements, JB and MS performed semen analysis, samples preparation and data collection, LR performed statistical analysis. All authors read and approved the final manuscript.
